# Edaravone, a Synthetic Free Radical Scavenger, Enhances Alteplase-Mediated Thrombolysis

**DOI:** 10.1155/2017/6873281

**Published:** 2017-11-10

**Authors:** Kiyoshi Kikuchi, Kentaro Setoyama, Ko-ichi Kawahara, Tomoka Nagasato, Takuto Terashi, Koki Ueda, Kazuki Nakanishi, Shotaro Otsuka, Naoki Miura, Hisayo Sameshima, Kazuya Hosokawa, Yoichiro Harada, Binita Shrestha, Mika Yamamoto, Yoko Morimoto-Yamashita, Haruna Kikuchi, Ryoji Kiyama, Chinatsu Kamikokuryo, Salunya Tancharoen, Harutoshi Sakakima, Motohiro Morioka, Eiichiro Tanaka, Takashi Ito, Ikuro Maruyama

**Affiliations:** ^1^Division of Brain Science, Department of Physiology, Kurume University School of Medicine, Kurume, Japan; ^2^Department of Neurosurgery, Kurume University School of Medicine, Kurume, Japan; ^3^Department of Systems Biology in Thromboregulation, Kagoshima University Graduate School of Medical and Dental Science, Kagoshima, Japan; ^4^Department of Pharmacology, Faculty of Dentistry, Mahidol University, Bangkok, Thailand; ^5^Division of Laboratory Animal Science, Natural Science Center for Research and Education, Kagoshima University, Kagoshima, Japan; ^6^Department of Biomedical Engineering, Laboratory of Functional Foods, Osaka Institute of Technology, Osaka, Japan; ^7^Research Institute, Fujimori Kogyo Co., Yokohama, Kanagawa, Japan; ^8^Course of Physical Therapy, School of Health Sciences, Faculty of Medicine, Kagoshima University, Kagoshima, Japan; ^9^Department of Veterinary Science, Laboratory of Diagnostic Imaging, Faculty of Agriculture, Kagoshima University, Kagoshima, Japan; ^10^Department of Restorative Dentistry and Endodontology, Kagoshima University Graduate School of Medical and Dental Science, Kagoshima, Japan; ^11^Department of Psychosomatic Internal Medicine, Kagoshima University Graduate School of Medical and Dental Science, Kagoshima, Japan; ^12^School of Health Sciences, Faculty of Medicine, Kagoshima University, Kagoshima, Japan; ^13^Department of Emergency and Critical Care Medicine, Kagoshima University Graduate School of Medical and Dental Science, Kagoshima, Japan

## Abstract

The combination of alteplase, a recombinant tissue plasminogen activator, and edaravone, an antioxidant, reportedly enhances recanalization after acute ischemic stroke. We examined the influence of edaravone on the thrombolytic efficacy of alteplase by measuring thrombolysis using a newly developed microchip-based flow-chamber assay. Rat models of embolic cerebral ischemia were treated with either alteplase or alteplase-edaravone combination therapy. The combination therapy significantly reduced the infarct volume and improved neurological deficits. Human blood samples from healthy volunteers were exposed to edaravone, alteplase, or a combination of alteplase and edaravone or hydrogen peroxide. Whole blood was perfused over a collagen- and thromboplastin-coated microchip; capillary occlusion was monitored with a video microscope and flow-pressure sensor. The area under the curve (extent of thrombogenesis or thrombolysis) at 30 minutes was 69.9% lower in the edaravone-alteplase- than alteplase-treated group. The thrombolytic effect of alteplase was significantly attenuated in the presence of hydrogen peroxide, suggesting that oxidative stress might hinder thrombolysis. D-dimers were measured to evaluate these effects in human platelet-poor plasma samples. Although hydrogen peroxide significantly decreased the elevation of D-dimers by alteplase, edaravone significantly inhibited the decrease. Edaravone enhances alteplase-mediated thrombolysis, likely by preventing oxidative stress, which inhibits fibrinolysis by alteplase in thrombi.

## 1. Introduction

Alteplase is the most effective and frequently used recombinant tissue plasminogen activator (tPA) for thrombolysis in patients with acute ischemic stroke (AIS) [1]. Tissue plasminogen activators are enzymes that catalyze the conversion of plasminogen to plasmin [2].

Edaravone (Radicut®; Mitsubishi Tanabe Pharma Corporation, Osaka, Japan) is a low-specificity antioxidant that scavenges various free radicals. Edaravone exhibits neurovascular protective effects against apoptosis, necrosis, edema, and inflammatory cytokines [3–9].

Several clinical trials have shown that edaravone-alteplase combination therapy is more effective than alteplase alone in patients with AIS [1, 10–13]. Kimura et al. [14] presumed that these therapeutic effects occurred because edaravone protected the endothelium from ischemic injury, which increased the endogenous tPA levels and promoted early recanalization [14–16]. However, the currently available global clinical evidence for the efficacy of edaravone is inadequate, and more basic evidence for the efficacy of this drug in combination with alteplase is needed.

In the ischemic brains of rats, edaravone prevents endothelial cell damage and blood-brain barrier disruption [17, 18]. These *in vivo* studies showed that edaravone-alteplase combination therapy was more effective than alteplase alone in rats with occlusion of the middle cerebral artery by an intraluminal nylon filament [17, 18]. However, no reports have described the establishment of a model of thromboembolic clot-induced cerebral ischemia, which would more accurately reflect clinical morbid conditions. Therefore, we used a model of thromboembolic clot-induced cerebral ischemia *in vivo*.

A recent experimental study showed that the thrombus volume was significantly lower with edaravone-alteplase combination treatment than with alteplase alone in a model of helium-neon laser-induced thrombosis of rat mesenteric microvessels [19]. Edaravone is considered to protect the endothelium, prevents new thrombus formation by enhancing the expression of endothelial nitric oxide synthase, improves nitric oxide release, and inhibits the expression of selectin [20]. An increased nitric oxide level leads to vasorelaxation, and downregulation of selectin suppresses platelet adhesion, platelet aggregation, and leukocyte adhesion [21]. Therefore, edaravone is expected to accelerate early recanalization. However, the effects of edaravone on the blood itself, not on endothelial cells, are unknown. Current *in vitro* assays of fibrinolytic reactions, such as clot-lysis tests, thromboelastography, and rotational thromboelastometry, are generally performed in the absence of blood flow; this limits their relevance to pathologic arterial thrombosis or physiological hemostasis [22, 23]. To overcome the limitations associated with animal models and static *in vitro* assays for assessing fibrinolysis, Hosokawa et al. [24] speculated that evaluating fibrin-rich platelet thrombus formation under shear flow could be a useful model for studying thrombolytic processes in the arterial circulation.

The aim of the present study was to examine the mechanism by which edaravone promotes alteplase-mediated thrombolysis *in vitro* in human blood donated by healthy volunteers. We used the newly developed Total Thrombus-formation Analysis System (T-TAS®; Fujimori Kogyo Co., Ltd., Tokyo, Japan) to quantify thrombolysis in blood exposed to edaravone, alteplase, or alteplase and edaravone. We also examined the influence of hydrogen peroxide (H_2_O_2_) on alteplase-induced thrombolysis. Finally, we measured the concentration of D-dimers, which are fibrin degradation products, in platelet-rich plasma (PRP) sump solutions collected after the T-TAS assay to determine whether edaravone-alteplase combination therapy inhibits thrombogenesis or promotes thrombolysis.

## 2. Materials and Methods

The experimental protocol was approved by the Institutional Animal Care and Use Committee of Kagoshima University (Kagoshima, Japan). The study protocol was approved by the local ethics committee of Kagoshima University, and written informed consent was obtained from all individuals prior to their participation.

### 2.1. Rat Model of Thromboembolic Ischemia

Thromboembolic ischemia of the middle and posterior cerebral arteries was induced in 8-week-old male Sprague–Dawley rats weighing 290 to 310 g as previously described [25], with some modifications. Anesthesia was induced and maintained with 2.5% to 3.0% isoflurane inhalation. After establishment of anesthesia, the rats were placed on the operating table in the supine position. The rectal temperature was kept at 37°C ± 1°C from the start of anesthesia until awakening. Initially, we performed a left femoral 1 cm skin incision, inserted a 0.7 × 1.9 mm 24-gauge catheter (Angiocath®; Becton Dickinson Co., Fukushima, Japan) into the left femoral artery under a microscope, and withdrew 0.15 ml of arterial blood. This blood was injected into a 1.5 ml microtube containing 10 units of thrombin (Sawai Pharmaceutical, Osaka, Japan) in 50 *μ*l of saline and kept at room temperature for 30 min. Centrifugation at 2800 rpm was performed for 2 min, and the supernatant serum was discarded. The clot was suctioned into 4-French polyvinyl chloride tubes (Atom Extension Tube; Atom Medical, Tokyo, Japan) with a 1ml disposable syringe (Termosyringe®; Terumo Co., Tokyo, Japan). Under an operating microscope, the left common, external, and internal carotid arteries were exposed through a midline incision. The external carotid artery was ligated, coagulated, and cut down just proximal to the lingual and maxillary artery branches. All other branches of the external carotid artery were coagulated and transected. The internal carotid artery was then isolated to avoid damage to the vagus nerve. The pterygopalatine artery was ligated at its origin. The internal and common carotid arteries were clamped with small aneurysm clips. A 24-gauge catheter (SURFLO Flash®; Terumo Co.) combined with a 1 ml syringe was inserted into the internal carotid artery via a small incision in the external carotid artery stump. The clot was pushed into the internal carotid artery via a 24-gauge catheter by means of a straightened 1 mm diameter paper clip. The thrombus occluded the distal internal carotid artery, the proximal portion of the anterior cerebral artery, the middle cerebral artery, and the posterior cerebral arteries. To evaluate the infarction volume, neuromotor function, and hemorrhagic transformation at 24 h, the 5 mm clot (volume of 3.6 mm^3^) was pushed into the internal carotid artery (*n* = 8 per group). After withdrawing the catheter, the external carotid artery was ligated. The temporary clip was withdrawn, and the internal carotid artery blood flow recovered.

We did not use a cerebral blood flow (CBF) monitor. Shimamura et al. [25] reported that a CBF monitor is not indispensable for this model because other surgical manipulations can be performed to establish whether brain injury and/or changes in intracranial pressure have been avoided. Additionally, dissection of the temporal muscle causes masticatory dysfunction, leading to inadequate nutrition. Instead, we used only rats with a neurological score of 3 or 4 after awakening. The rats were evaluated for neurological deficits after awakening and at 24 h after thromboembolism. A neurological grading system with a 5-point motor function scale (0–4) was used as previously described [26]. The scale was as follows: 0 = no apparent deficits, 1 = right forelimb flexion, 2 = decreased right forelimb grip when tail is pulled, 3 = spontaneous movement in all directions with right circling only when tail is pulled, and 4 = spontaneous right circling.

### 2.2. Drug Treatment

Three groups of rats were studied as follows: the vehicle-injected control group, alteplase group, and edaravone-alteplase group. Edaravone and alteplase were provided by Mitsubishi Tanabe Pharma Corporation. Immediately after thromboembolism, 6 mg/kg of edaravone was administered over a 20 min period via a jugular vein catheter using an infusion pump. At 20 min after thromboembolism, 3 mg/kg of alteplase was administered shortly after edaravone administration. The alteplase group received vehicle instead of edaravone, followed by alteplase treatment. The control group received injections of vehicle instead of edaravone and alteplase. The dose and timing of administration of edaravone and alteplase were determined via preliminary experiments to maximize the effect of edaravone [5]. Therefore, the dose of alteplase was lower than that in previous reports using 10 mg/kg [17, 18].

### 2.3. Measurement of Infarct Volume

After venous blood sampling, the rats were killed and their brains excised 24 h after thromboembolism as previously described [26]. Physiological saline was transcardially perfused before decapitation. The brain was carefully removed and cut into six 2 mm thick coronal sections from the frontal tip using a brain slicer. The slices were then immersed in a 1% solution of 2,3,5-triphenyltetrazolium chloride in phosphate buffered saline (pH 7.4) at 37°C for 10 min. After staining, the sections were scanned to determine the ischemic infarct volume. The infarctions were measured using Scion Image software versus Beta 4.0.3 (Scion Corp., Frederick, MD). The total infarct area (mm^3^) was multiplied by the thickness of the brain sections to obtain the infarct volume. Additionally, the presence of visible hematomas or hemorrhagic transformation was recorded.

### 2.4. Rat Blood Samples and Laboratory Data

At 24 h after thromboembolism, the rats were deeply anesthetized via an intraperitoneal injection of 4% chloral hydrate (10 ml/kg). Blood samples were collected from the axillary vein.

Adverse drug reactions such as renal and hepatic disorders are occasionally observed during edaravone treatment in >5% of patients [27]. To evaluate adverse drug reactions including renal and hepatic disorders, the serum aspartate transaminase, serum alanine transaminase, blood urea nitrogen, and serum creatinine levels were measured by an enzymatic method using a Fuji DRI-CHEM Slide Kit (Fujifilm Medical, Tokyo, Japan).

### 2.5. Human Blood Samples

Blood samples from 10 healthy, fasting Japanese volunteers (mean age, 40.3 ± 12.7 years) were collected in plastic tubes containing 3.2% sodium citrate (Terumo Co.). None of the volunteers had taken antithrombotic drugs within 2 weeks of the study. One volunteer regularly took an over-the-counter fish oil supplement (docosahexaenoic acid 300 mg/day, eicosapentaenoic acid 100 mg/day). Normal ranges for the T-TAS analysis have not yet been defined, but the T-TAS findings of all volunteers' samples lay within 95% of the median of 123 healthy Japanese individuals who participated in our preliminary study (data not shown). Platelet-rich plasma was prepared by centrifugation at 800 rpm for 15 min, and platelet-poor plasma (PPP) was prepared by centrifugation at 3000 rpm for 15 min.

In the experiments using whole blood and PRP samples, we selected the final concentration of alteplase (500 IU/ml) and edaravone (3 *μ*M) based on half the maximum concentration after administration to Japanese patients with AIS [5, 28]. In the experiments using PPP samples, we selected the final concentration of alteplase (50, 100, and 250 IU/ml) and edaravone (6 and 60 nM) based on the findings of a preliminary experiment. The final concentrations of vitamin C (6 *μ*M), vitamin E (120 nM), and NAC (6 *μ*M) were selected based on a preliminary experiment and previous reports and were expected to have efficacy equivalent to that of edaravone [29]. The final concentration of H_2_O_2_ (100 *μ*M) was based on a previous report, recognizing that it is difficult to estimate the local concentration of reactive oxygen species (ROS) around the intravascular thrombi during AIS [5]. Vitamins C and E were obtained from Kanto Kagaku Co., Ltd. (Tokyo, Japan), NAC was obtained from Wako Pure Chemicals (Osaka, Japan), and H_2_O_2_ was obtained from Sigma-Aldrich Japan (Tokyo, Japan).

Samples from six healthy, fasting volunteers (mean age, 35.8 ± 7.6 years) were further selected for the experiments in which specimens were exposed to H_2_O_2_. These 6 volunteers were selected from the original cohort of 10 because they had a normal response to alteplase. Of the remaining volunteers, one was a nonresponder, two were incomplete responders, and one was an over-responder.

### 2.6. T-TAS

The thrombolytic effects of alteplase, edaravone, and the combination of alteplase and edaravone were compared with the controls under flow conditions using T-TAS in whole blood and PRP. To quantify thrombogenesis and thrombolysis under flow conditions, the T-TAS assay was performed as previously described [24]. Thrombogenesis and thrombolysis were observed in the microchip using a built-in light microscope. An antioxidant (edaravone, vitamin C or E, or NAC) was added to the blood samples 10 min before the addition of alteplase. Hydrogen peroxide was added immediately after the addition of alteplase. As soon as alteplase or H_2_O_2_ had been administered, each sample was perfused over a microchip coated with collagen and tissue thromboplastin to promote thrombosis at a flow rate of 4 *μ*l/min, corresponding to an initial wall shear rate of 240 per second.

### 2.7. Measurement of D-Dimer Concentration in Sump Solutions of T-TAS

T-TAS sump solutions were prepared by diluting the analyzed pooled PRP samples at a 1 : 25 ratio in ethylenediaminetetraacetic acid followed by centrifugation at 800 rpm for 15 min. The concentration of D-dimers was also measured in the sump solution using an LPIA-NV7 instrument and RM73-752YLK solution (LSI Medience Corporation, Tokyo, Japan).

### 2.8. Fibrinolysis Assays

Fibrinolysis assays were performed to evaluate whether edaravone enhances thrombolysis by alteplase. Microplate-based fibrinolysis assays were performed at 37°C in flat-bottomed 96-well polystyrene plates (Corning; Sigma-Aldrich Japan) by monitoring turbidity changes (A_405_) using a VersaMax microplate reader (Molecular Devices Japan, Tokyo, Japan). Calcium ions accelerate the formation of a fibrin clot from fibrinogen in the presence of thrombin. One-hundred microliter aliquots of 30% human PPP pooled from all 10 volunteers and TBSTC (8 mM Tris at pH 7.4, 0.008% Tween-20, and 12 mM calcium chloride) were prepared in the presence or absence of alteplase (0, 50, 100, or 250 IU/ml) or edaravone (0, 6, or 60 nM; *n* = 5). The concentrations of edaravone had been determined in a preliminary experiment. Edaravone was added to the samples 10 min before alteplase.

### 2.9. Measurement of D-Dimer Concentration in Human PPP Samples

D-dimers were measured to determine whether edaravone attenuates the inhibition of alteplase-induced fibrinolysis by H_2_O_2_. Four-hundred microliter aliquots of 30% human PPP pooled from all 10 volunteers and TBSTC were prepared. After incubation at 37°C for 15 min, H_2_O_2_ (100 *μ*M), edaravone (60 nM), or vehicle was added. After incubation at 37°C for 10 min, alteplase (100 IU/ml), plasmin (250 *μ*g/ml), or vehicle was added. After fibrin deposition had occurred by incubating at 37°C for 10 min, aprotinin (400KIU/ml) was added. After centrifugation at maximum speed for 5 min, the concentration of D-dimers was measured using an ACL TOP automated analyzer (Instrumentation Laboratory, Bedford, MA) (*n* = 5).

### 2.10. Statistical Analysis

The neurological score, infarction volume, hemorrhagic transformation rate, and blood test data were analyzed using the Steel–Dwass method or Bonferroni–Dunn method as appropriate in multiple comparisons.

The area under the curve at 30 min (AUC30) was calculated to evaluate the extent of thrombogenesis or thrombolysis. The AUC30 represents the area under the flow-pressure curve (<80 kPa) 30 min after the start of assay, as previously described [24]. The AUC30 was also used to quantify the impairment of thrombus formation when occlusion is not achieved during an assay. Comparison between two groups was performed using the paired *t*-test or Wilcoxon signed-rank test as appropriate.

Absorbance data were analyzed by repeated-measures two-way analysis of variance followed by Bonferroni's test.

Data are presented as mean ± standard deviation unless otherwise indicated. Differences with *P* < 0.05 were considered statistically significant. All analyses were performed using SPSS Statistics (version 20; IBM Corp., Armonk, NY).

## 3. Results

### 3.1. Edaravone-Alteplase Combination Reduces Infarct Volume and Improves Neuromotor Function in Rats

**Figure 1:**
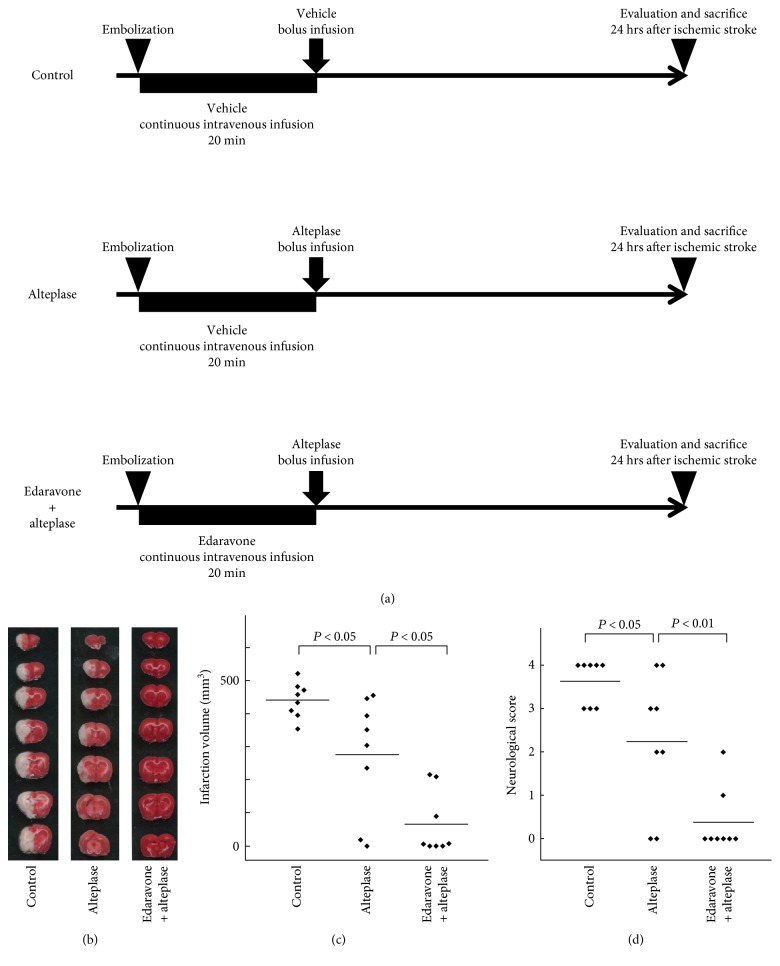
Effect of edaravone-alteplase combination therapy in a rat model of thromboembolic clot-induced cerebral ischemia. (a) The experimental groups included the vehicle-injected control group, alteplase group, and edaravone-alteplase combination group (*n* = 8 per group). Rats were killed 24 hours after establishment of cerebral ischemia. (b) Representative figures of 2,3,5-triphenyltetrazolium chloride-stained brain sections of rats. Normal brain tissue stains deep red, and ischemic lesions are white (unstained). (c) Infarct volume in the control, alteplase, and edaravone-alteplase groups. (d) Neurological score in the control, alteplase, and edaravone-alteplase groups. The horizontal lines represent the mean values.

We evaluated our rat thromboembolic stroke model, in which the distal internal carotid artery, proximal portion of the anterior cerebral artery, middle cerebral artery, and posterior cerebral arteries were occluded by autologous thrombi (Figure 1(b)). We compared the vehicle-injected control group, alteplase group, and edaravone-alteplase combination group, which allowed us to investigate the therapeutic effects of edaravone-alteplase combination therapy (Figure 1(a)).

We did not use a CBF monitor according to a previous report [25]. The neurological score of all rats after awakening was 3 or 4, and the differences among the three groups of rats that were assigned to the different treatments were not statistically significant (Table 1). Therefore, we were able to induce cerebral ischemia without using a CBF monitor.

We then evaluated the infarct volume, neuromotor function, hemorrhagic transformation, and adverse drug reactions at 24 h after ischemia in the three groups. Compared with the controls, alteplase significantly reduced the infarct volume (*P* < 0.05) (Figures 1(b) and (c)). However, the infarct volume was further significantly decreased in rats receiving edaravone-alteplase (*P* < 0.05) (Figures 1(b) and (c)). The neurologic score in rats receiving alteplase was significantly better than that in the controls (*P* < 0.05) (Figure 1(d)). Additionally, the rats treated with edaravone-alteplase showed significantly better neurologic scores than the rats treated with alteplase (*P* < 0.01) (Figure 1(d)). Meanwhile, the rate of hemorrhagic transformation tended to be lower in the edaravone-alteplase group than in the alteplase group. However, the differences were not statistically significant among the three groups (Table 1).

Adverse drug reactions, including renal and hepatic disorders, were not apparent because the serum aspartate transaminase, serum alanine transaminase, blood urea nitrogen, and serum creatinine levels were not significantly different among the three groups (Table 1).

In conclusion, edaravone synergized with acute alteplase treatment in this experimental thrombotic stroke model.

### 3.2. Characteristics of Blood Samples Obtained from Healthy Volunteers

The mean erythrocyte, leukocyte, and platelet counts of whole blood and PRP samples are shown in Table 2. These lay within the normal ranges for healthy Japanese individuals.

### 3.3. Edaravone Enhances Alteplase-Mediated Thrombolysis in Human Whole Blood

**Figure 2:**
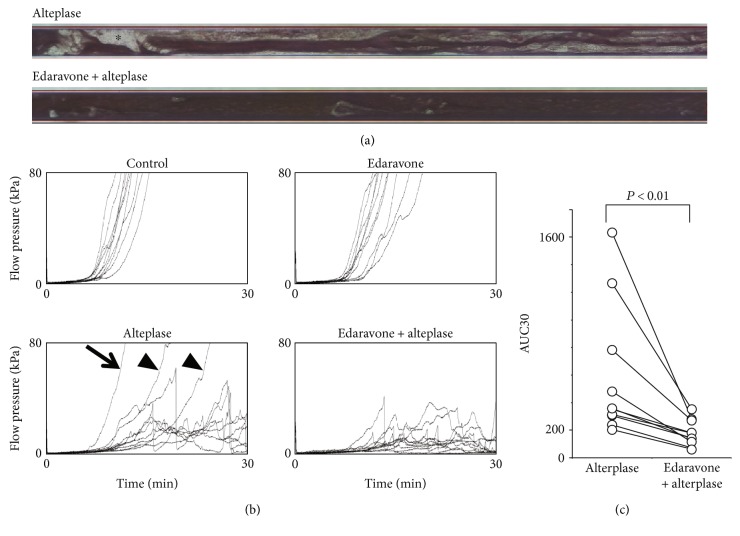
Effect of edaravone on alteplase-induced thrombolysis under flow conditions in whole blood. (a) Typical still videomicroscopy images of thrombogenesis and thrombolysis over 27 to 28 min in samples exposed to alteplase or edaravone-alteplase. The asterisk (white area) indicates thrombi. (b) Flow-pressure curves in the control, edaravone, alteplase, and edaravone-alteplase groups (*n* = 10). The arrow indicates an alteplase nonresponder, and the arrowhead indicates incomplete responders. (c) AUC30 in the alteplase and edaravone-alteplase groups. AUC30: area under the curve at 30 min.

To clarify the mechanism of the synergistic effects of edaravone in the animal model (Figure 1), we next evaluated whether edaravone enhances alteplase-mediated thrombolysis under flow conditions using the T-TAS in human whole blood (Figure 2). After perfusion had started, plentiful small white thrombi were observed adhering to the coated surface. The thrombi gradually increased in size and merged with each other, leading to capillary occlusion in 9 to 10 min in the control and edaravone groups (data not shown). In the alteplase and edaravone-alteplase groups, capillary occlusion occurred at 18 to 19 min. However, in the edaravone-alteplase group, the thrombi had dissolved within 27 to 28 min (Figure 2(a)). Treatment with alteplase alone had a limited effect on thrombus firmness, but in the presence of edaravone, thrombus firmness diminished as evidenced by the frequent collapse of thrombi.

Thrombus formation caused microcapillary occlusion in all control samples not exposed to alteplase, and thrombolysis was evident in the majority of samples exposed to alteplase. However, the perfused capillary became completely occluded in three samples exposed to alteplase (one in which there was no response to alteplase [10%] and two in which there was an incomplete response [20%]). An exaggerated response to alteplase was observed in one sample (10%). In alteplase nonresponders, thrombolysis as evidenced by reduced thrombus firmness and the frequent collapse of thrombi was observed in samples to which both edaravone and alteplase had been added. The sample provided by the volunteer who regularly took the fish oil supplement exhibited a normal response to alteplase.

Microcapillary occlusion occurred in all control samples and the edaravone group and in three samples in the alteplase group but in none of the samples in the edaravone-alteplase group (Figure 2(b)). We observed a characteristic periodic flow-pressure pattern that reflected the collapse of thrombi, consistent with the lack of microcapillary occlusion in the edaravone-alteplase group. The synergistic effect of edaravone-alteplase combination therapy was evaluated by calculating the AUC30, which was significantly lower in the edaravone-alteplase group than in the alteplase group (*P* < 0.01) (Figure 2(c)). There was no significant difference in the AUC30 between the control group (1574.9 ± 108.8) and edaravone group (1495.2 ± 153.7) (*P* = 0.997). In conclusion, edaravone enhanced alteplase-mediated thrombolysis in human whole blood.

### 3.4. Other Antioxidants Enhance Alteplase-Mediated Thrombolysis in Human Whole Blood

**Figure 3:**
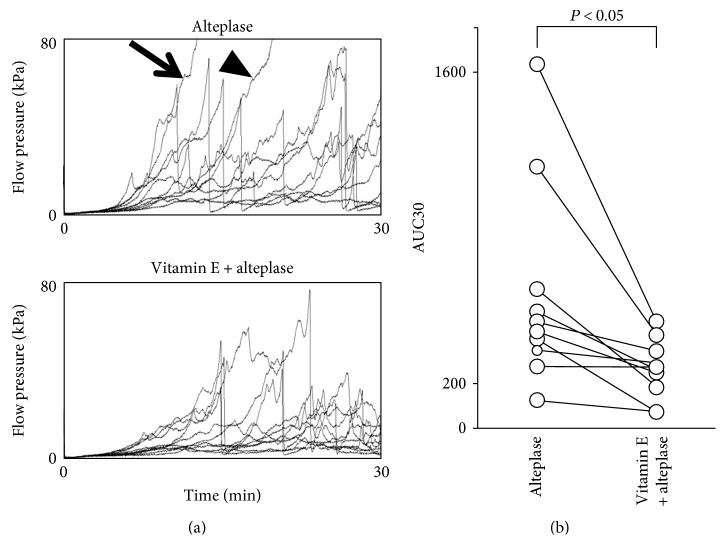
Effect of vitamin E on alteplase-induced thrombolysis under flow conditions in whole blood. (a) Flow-pressure curves in the alteplase and vitamin E-alteplase groups (*n* = 10). The arrow indicates an alteplase nonresponder, and the arrowhead indicates an incomplete responder. (b) AUC30 in the alteplase and vitamin E-alteplase groups. AUC30: area under the curve at 30 min.

Thrombolysis enhancement may be an edaravone-specific effect in human whole blood (Figure 2). Whether antioxidants other than edaravone also have a thrombolysis-enhancing effect is unknown. We examined and compared the synergistic effect of alteplase with other general antioxidants under flow conditions using the T-TAS in whole blood (Figure 3). Microcapillary occlusion occurred in two of the samples in the alteplase group but in none of the samples in which vitamin E was coadministered with alteplase (Figure 3(a)). In the alteplase-vitamin E group, the AUC30 was significantly lower than that in the alteplase alone group (*P* < 0.05) (Figure 3(b)). The AUC30 was also significantly lower in the alteplase-NAC group (316.8 ± 246.1) than the alteplase alone group (601.6 ± 457.4) (*P* < 0.05). However, there was no significant difference in the AUC30 between the alteplase-vitamin C group (469.0 ± 500.1) and the alteplase alone group (*P* = 0.334). These findings confirm that some general antioxidants have a thrombolysis-enhancing effect similar to that of edaravone.

### 3.5. Reactive Oxygen Species Inhibit Alteplase-Mediated Thrombolysis in Human Whole Blood

**Figure 4:**
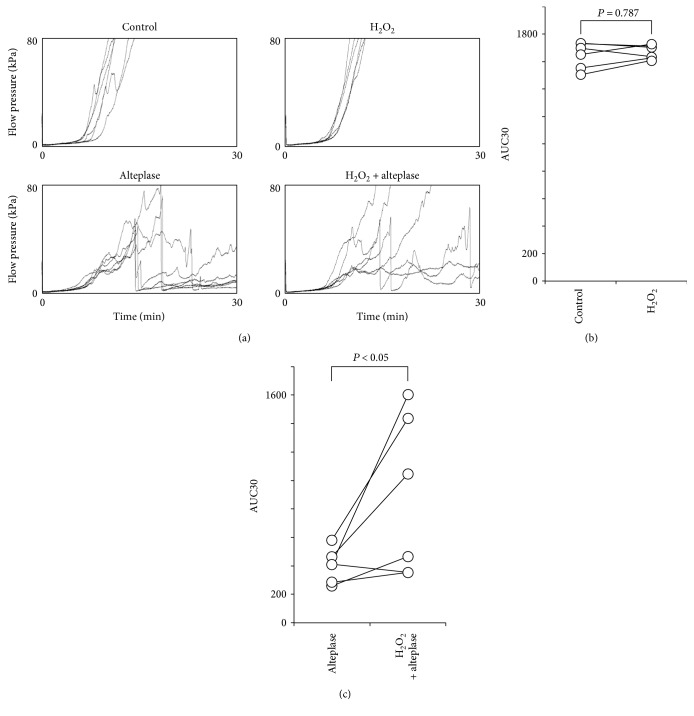
Effect of H_2_O_2_ on alteplase-induced thrombolysis under flow conditions in whole blood. (a) Flow-pressure curves in the control, H_2_O_2_, alteplase, and H_2_O_2_-alteplase groups (*n* = 6). (b) AUC30 in the control and H_2_O_2_ groups. (c) AUC30 in the alteplase and H_2_O_2_-alteplase groups. AUC30: area under the curve at 30 min; H_2_O_2_: hydrogen peroxide.

Edaravone and other antioxidants (vitamin E and NAC) might enhance alteplase-mediated thrombolysis in whole blood (Figure 3). ROS may be the key molecular targets of these antioxidants, including edaravone. We examined whether ROS such as H_2_O_2_ affect thrombogenesis or thrombolysis in whole blood under flow conditions using the T-TAS (Figure 4). Addition of 100 *μ*M H_2_O_2_ to samples exposed to alteplase resulted in microcapillary occlusion in a greater proportion of samples than those exposed to alteplase alone (Figure 4(a)). The AUC30 in the H_2_O_2_ alone group was broadly comparable with that in controls (*P* = 0.787) (Figure 4(b)), but the AUC30 was significantly higher in the alteplase-H_2_O_2_ group than in the alteplase alone group (*P* < 0.05) (Figure 4(c)). Therefore, ROS inhibited thrombolysis but did not induce thrombogenesis at this concentration (100 *μ*M H_2_O_2_).

### 3.6. Edaravone Enhances Alteplase-Mediated Thrombolysis in Human Plasma Components

**Figure 5:**
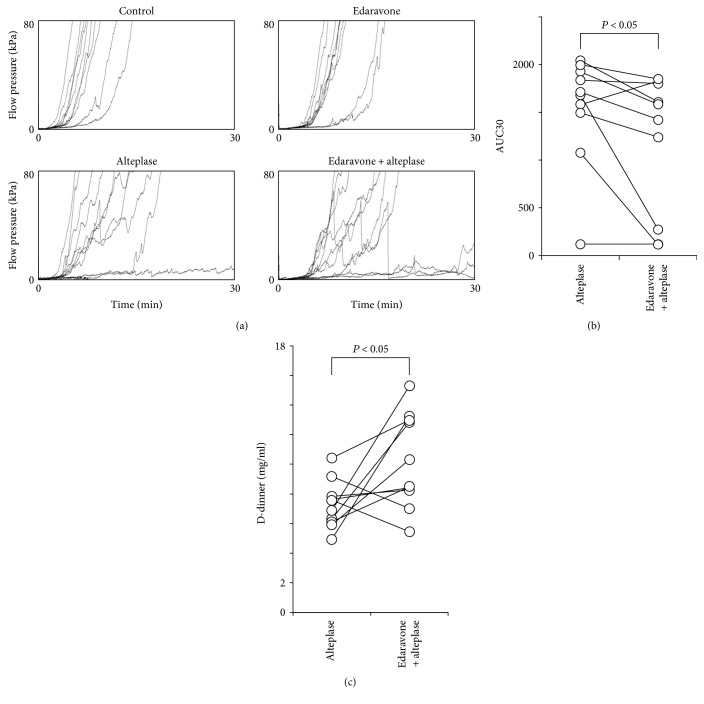
Effect of edaravone on alteplase-induced thrombolysis in PRP. (a) Flow-pressure curves in the control, edaravone, alteplase, and edaravone-alteplase groups in PRP under flow conditions (*n* = 10). (b) AUC30 in the alteplase and edaravone-alteplase groups in PRP. (c) D-dimer concentration in sump solutions after measurement using the Total Thrombus-formation Analysis System in PRP in the alteplase and edaravone-alteplase groups (*n* = 10). AUC30: area under the curve at 30 min; PRP: platelet-rich plasma.

Pertaining to which components of whole blood are affected by edaravone is unclear. Comparison of whole blood and PRP samples revealed negligible numbers of erythrocytes and leukocytes in PRP samples (Table 2). Therefore, which blood components are affected by edaravone may become evident by comparing its effect between whole blood and PRP samples. Therefore, we examined the thrombolytic effects of alteplase, edaravone, and their combination under flow conditions using the T-TAS in PRP (Figures 5(a) and (b)). Exposure of PRP (Table 2) to alteplase, edaravone, or edaravone-alteplase under flow conditions reduced the proportion in which microcapillary occlusion occurred (Figure 5(a)). The synergistic effect of alteplase and edaravone was reflected by a significantly lower AUC30 in the edaravone-alteplase than alteplase group (*P* < 0.05) (Figure 5(b)). Therefore, the thrombolysis-enhancing effect of edaravone was confirmed in PRP samples.

The thrombolysis-enhancing effect of edaravone was confirmed objectively and quantitatively in whole blood and PRP (Figures 2, 5, and 5). However, whether a low AUC on the T-TAS inhibits thrombogenesis or enhances thrombolysis remains unclear. In one ex vivo study, alteplase dissolved retrieved human cerebral thromboemboli and induced D-dimers (i.e., fibrin degradation products) and minimum protein fragment [30]. No studies have determined whether edaravone enhances induction of D-dimer release by alteplase. Therefore, we evaluated D-dimers in PRP sump solutions after measuring PRP samples using the T-TAS to confirm the thrombolysis-enhancing effect of edaravone in healthy human blood samples (Figure 5(c)). The mean concentration of D-dimers in the PRP sump solutions collected after the T-TAS assay was significantly higher in the edaravone-alteplase group (10.2 ± 3.2 mg/ml) than in the alteplase alone group (7.3 ± 1.6 mg/ml) (*P* < 0.05) (Figure 5), demonstrating an inverse relationship between the AUC30 measured by the T-TAS and the D-dimer concentration. The D-dimer concentrations in all control samples (100%) were <0.03 mg/ml, which is below the measurable range. In the edaravone alone group, the D-dimer concentration was <0.03 mg/ml in 8 of 10 samples (80.0%) and 0.03 and 1.51 mg/ml in the remaining samples. These findings suggest that thrombolysis as evaluated by the T-TAS is strongly correlated with the D-dimer level.

**Figure 6:**
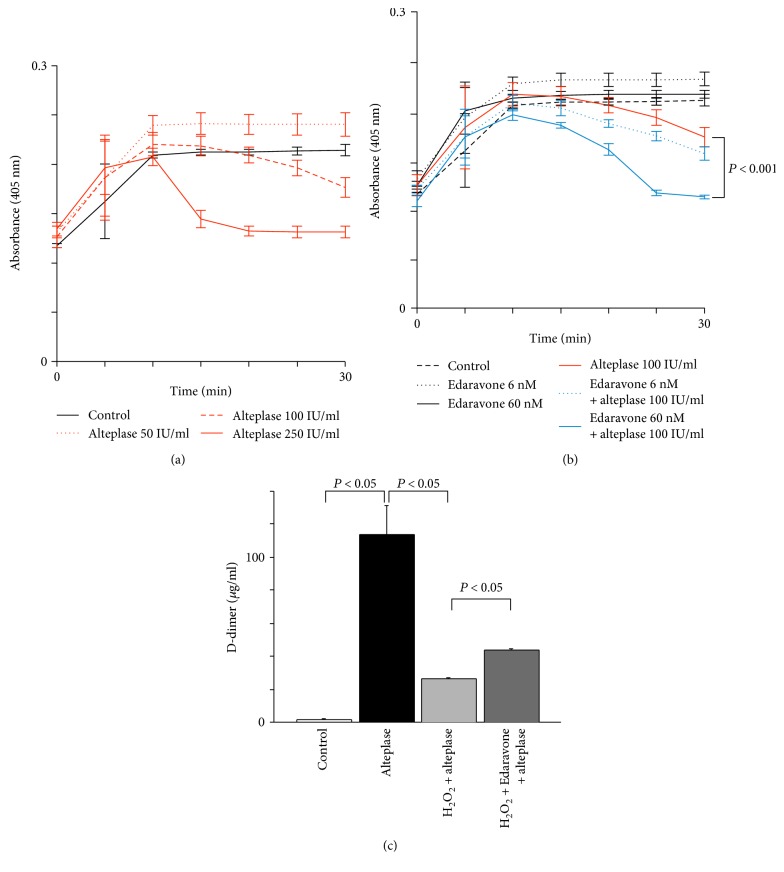
Effect of edaravone on alteplase-induced fibrinolysis in PPP. (a) Effects of alteplase (50, 100, or 250 IU/ml) in pooled platelet-poor plasma (*n* = 5). Error bars represent standard deviation. (b) Effects of edaravone (6 or 60 nM), alteplase (100 IU/ml), or their combination on alteplase-induced thrombolysis in pooled PPP (*n* = 5). Error bars represent standard deviation. (c) Measurement of D-dimer concentration to evaluate effects of edaravone (60 nM) on H_2_O_2_ (100 *μ*M)-inhibited fibrinolysis by alteplase (100 IU/ml) in pooled PPP (*n* = 5). Error bars represent standard deviation. PPP: platelet-poor plasma; H_2_O_2_: hydrogen peroxide.

The synergistic effect of edaravone-alteplase combination therapy was confirmed objectively by the AUC30 and quantitatively in both whole blood and PRP (Figures 2 and 5). However, the effects of edaravone-alteplase combination therapy on either platelets or plasma are unclear because both platelets and plasma are present in both whole blood and PRP samples. However, almost no platelets are present in PPP samples. Therefore, pertaining to which blood components are affected by edaravone may be clarified by comparing its effect separately in whole blood samples, PRP samples, and PPP samples. PPP samples could not be evaluated because the capillaries did not become occluded in the T-TAS assay. The lack of platelets in PPP meant that the microcapillaries did not become occluded. Therefore, we examined the effects of alteplase, edaravone, and their combination on fibrinolysis in PPP using a method other than the T-TAS (Figure 6). In the alternative turbidity assay, exposure of PPP to alteplase (50, 100, or 250 IU/ml) caused dose-dependent fibrinolysis (Figure 6(a)). In PPP samples exposed to alteplase at 100 IU/ml, fibrinolysis was significantly enhanced by addition of edaravone at 60 nM (*P* < 0.001) (Figure 6(b)), but not by edaravone at 6 nM (*P* = 0.140). Therefore, the thrombolysis-enhancing effect of edaravone was confirmed in PPP samples.

The D-dimer concentration was significantly higher in PPP samples exposed to alteplase at 100 IU/ml than in controls (*P* < 0.05) (Figure 6(c)). Moreover, in samples exposed to alteplase at 100 IU/ml, the D-dimer concentration was significantly reduced by the addition of H_2_O_2_ at 100 *μ*M (*P* < 0.05) (Figure 6(c)). Nevertheless, in samples exposed to alteplase at 100 IU/ml and H_2_O_2_ at 100 *μ*M, the D-dimer concentration was significantly increased by the addition of edaravone at 60 nM (*P* < 0.05) (Figure 6(c)). These observations confirm the mechanism of the thrombolysis-enhancing effect of edaravone. ROS may inhibit alteplase-mediated thrombolysis, which is prevented by edaravone. Meanwhile, alteplase but not plasmin was enhanced by edaravone. In PPP samples exposed to plasmin (3.35 ± 2.08 *μ*g/ml) compared with plasmin and edaravone (2.03 ± 2.23 *μ*g/ml), the D-dimer concentration was not increased by the addition of edaravone.

## 4. Discussion

**Figure 7:**
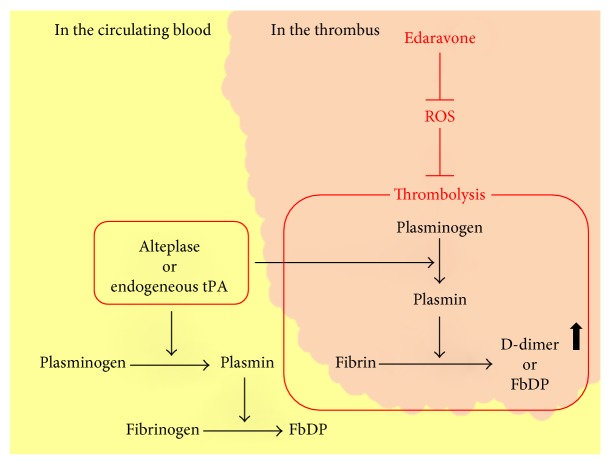
Schema showing the potential actions of edaravone against reactive oxygen species in thrombolytic pathways. Those in red indicate mechanisms suggested by the findings of our study. tPA: tissue plasminogen activator; FbP: fibrin degradation product; FgDP: fibrinogen degradation product.

The synergistic effects of edaravone-alteplase combination therapy have long been considered due to the neurovascular protective effect of edaravone targeting ROS and matrix metalloproteinase-9 [1, 17, 18]. However, we found that, although edaravone enhanced alteplase-mediated thrombolysis, edaravone alone neither attenuated thrombogenesis nor enhanced thrombolysis. This suggests that edaravone suppresses the inhibitory action of ROS on alteplase-mediated thrombolysis (Figure 7). Our results are in agreement with previous clinical studies [10–13].

The enhancing effect of edaravone against alteplase-mediated thrombolysis was confirmed in PRP, PPP, and whole blood. Whole blood contains leukocytes, unlike PRP or PPP. Moreover, H_2_O_2_ inhibited alteplase-mediated thrombolysis in both whole blood samples and PPP. Edaravone may enhance alteplase-mediated thrombolysis by inhibiting generation of ROS by platelets, plasma components, and leukocytes. Platelets are also reported to produce ROS and to release platelet-derived exosomes that in turn can generate ROS [31]. The effect of edaravone that we observed in PRP and PPP samples may reflect its activity against platelet- or exosome-derived ROS.

Although we were unable to identify the precise mechanism by which edaravone enhances the activity of alteplase, previous *in vitro* and *in vivo* studies revealed that tPA induces ROS and that ROS inhibit thrombolysis. Tissue plasminogen activators are Mac-1 (CD11b/CD18) ligands; Mac-1 mediates adhesion-dependent H_2_O_2_ production by human neutrophils [32]. Moreover, edaravone appears to ameliorate alteplase-induced oxidative stress in the rat brain, such as 4-hydroxy-2-nonenal and N-(hexanoyl)-lysine (lipid peroxidation markers), 8-hydroxy-2′-deoxyguanosine (a DNA oxidation marker), and advanced glycation end products (protein oxidation markers) [28]. Furthermore, ROS may induce plasminogen activator inhibitor-1 (PAI-1), while antioxidants abolish the induction of PAI-1 [33]. Therefore, the presence of an antioxidant, such as edaravone, may enhance thrombolysis by a pleiotropic mechanism.

Edaravone enhances the thrombolytic effect of alteplase; thus, edaravone-alteplase combination therapy may increase the incidence of adverse events such as intracerebral hemorrhage in patients treated for AIS. We found that edaravone had no thrombolytic effect when administered alone in coagulating blood. Edaravone may inhibit hemorrhagic transformation. This may be explained by a reduction in the frequency of adverse events caused by alteplase: the rate of development of symptomatic intracerebral hemorrhage attributed to alteplase infusion is reportedly negatively correlated with the rate of combined treatment with edaravone according to several clinical trials. [1]. This was also evident in our study. We found that alteplase but not plasmin was enhanced by edaravone. The fibrin-binding affinity of alteplase can be impaired by exposure to ROS, and the characteristic advantage of thrombus selectivity of alteplase in both spontaneous thrombolysis and thrombolytic therapy may be diminished in environments where ROS are plentiful [34]. Plasminogen exists in both circulating blood and thrombi, and plasmin degrades both fibrinogen and fibrin. Therefore, alteplase may activate plasminogen in the circulating blood rather than in thrombi under ROS-rich conditions. This would result in the production of FDP from fibrinogen in the circulating blood, while the fibrin in thrombi may decompose to a lesser degree. Edaravone-alteplase combination therapy may increase the affinity of alteplase for fibrin and cause plasmin activation to be selective for thrombi. Therefore, fibrinogen degradation associated with plasminogen activation in the circulating blood, which is not in thrombi, is less likely to occur. This may reduce the risk of bleeding tendencies. Our results are in agreement with previous clinical studies [1]. Edaravone may enhance thrombolysis and inhibit adverse events such as intracerebral hemorrhage because edaravone enhances alteplase-mediated thrombolysis, likely by preventing inhibition of alteplase-induced fibrinolysis by oxidative stress in thrombi (Figure 7).

Edaravone is not currently licensed for use in Western countries. A new formulation and dosing regimen was recently evaluated in Finland, the Netherlands, and the United Kingdom, which may promote the use of edaravone more widely throughout the European Union [35]. Meanwhile, the US Food and Drug Administration approved edaravone for the treatment of amyotrophic lateral sclerosis in 2016 [36, 37]. Therefore, a study of edaravone-alteplase combination therapy in Western countries might also be carried out in the near future. The outcomes of future studies may show whether such combination therapy is a breakthrough treatment for AIS.

### 4.1. Study Limitations

In clinical practice, alteplase is administered after thrombosis and blood vessel occlusion. In the present experiment, however, it was added before thrombosis was initiated. Furthermore, the reduction in the AUC30 in the T-TAS assay that we observed does not illuminate whether thrombogenesis is being inhibited or thrombolysis is being promoted. However, the elevated D-dimer concentration that we found in the sump solutions suggests that thrombolysis (lysis of fibrin clots) occurred after thrombogenesis, implying that edaravone was enhancing alteplase-mediated thrombolysis in the T-TAS assay.

Direct determination of oxidative stress such as that caused by ROS or free radicals is not easy. Moreover, obtaining high reproducibility is difficult because the circadian variation of endogenous tPA or PAI-1 is intense, and these agents are very unstable. Meanwhile, the direct binding affinity of alteplase, H_2_O_2_, and edaravone against precipitated fibrin should be evaluated, but we could not perform such an evaluation on an Octet system (Pall ForteBio, Fremont, CA) by analysis method of interbiomolecule interaction.

## 5. Conclusions

We investigated the effect of edaravone on alteplase-induced thrombolysis using a rat model of thromboembolic clot-induced cerebral ischemia (severe cardioembolic cerebral infarction *in vivo*). Moreover, we examined the mechanism by which edaravone promotes alteplase-mediated thrombolysis *in vitro* in human blood donated by healthy volunteers using a newly developed microchip-based flow-chamber assay (the Total Thrombus-formation Analysis System) to perform a quantitative analysis under flow conditions. We showed that edaravone is an enhancer of alteplase, although previous reports have only shown the synergistic effect of edaravone as a neuroprotectant. The thrombolytic effect of alteplase was significantly attenuated in the presence of hydrogen peroxide, suggesting that oxidative stress might hinder thrombolysis. Edaravone alone did not influence thrombosis or thrombolysis. Edaravone enhances alteplase-mediated thrombolysis *in vitro*, likely by acting as an antioxidant to prevent free radical-related inhibition of alteplase activity on thrombi. Furthermore, edaravone significantly attenuated inhibition of alteplase-induced fibrinolysis by hydrogen peroxide as shown by the measurement of plasma D-dimers in human platelet-poor plasma.

## Figures and Tables

**Table 1:** Therapeutic Effects and Laboratory Data in the Rat Thromboembolic Ischemic Model.

	Control	Alteplase	Edaravone-alteplase combination
	*n*	*n*	*n*
*Total number of rats*			
	8	8	8
*Neurological score after awakening*			
3	5	4	3
4	3	4	5
*Hemorrhagic transformation*			
	0	2	0
*Blood test data*			
AST (U/L)	233.0 ± 241.5	182.5 ± 30.0	234.4 ± 125.1
ALT (U/L)	34.0 ± 11.8	34.4 ± 3.9	42.3 ± 19.2
BUN (mg/dl)	12.4 ± 1.3	13.2 ± 1.4	13.4 ± 1.9
Cr (mg/dl)	0.18 ± 0.07	0.18 ± 0.05	0.19 ± 0.07

Blood test data are shown as mean ± standard deviation. BUN: blood urea nitrogen; Cr: creatinine; AST: aspartate transaminase; ALT: alanine transaminase.

**Table 2:** Erythrocyte, leukocyte, and platelet counts in whole blood and platelet-rich plasma used in the Total Thrombus-formation Analysis System assay.

	Whole blood	Platelet-rich plasma
Erythrocyte count (×10^6^/*μ*l)	4.78 ± 0.42	0.01 ± 0.01
Leukocyte count (×10^3^/*μ*l)	5.78 ± 1.07	0.02 ± 0.01
Platelet count (×10^3^/*μ*l)	224 ± 4	313 ± 105

Values are shown as mean ± standard deviation.
